# C-Reactive Protein and Lymphocyte-to-Monocyte Ratio Predict Recurrence in Stage III Melanoma Patients with Microscopic Sentinel Lymph Node Metastasis

**DOI:** 10.3390/cancers15030702

**Published:** 2023-01-23

**Authors:** Viktoria Anna Sophie Schildbach, Susanne Horn, Guillermo Hidalgo-Gadea, Wibke Johannis, Cornelia Mauch, Cindy Franklin

**Affiliations:** 1 Department of Dermatology and Venereology, Faculty of Medicine and University Hospital Cologne, University of Cologne, 50935 Cologne, Germany; 2 Center for Integrated Oncology (CIO), Aachen Bonn Cologne Düsseldorf, 50935 Cologne, Germany; 3 Medical Faculty of the University Leipzig, Rudolf-Schönheimer-Institute of Biochemistry, 04109 Leipzig, Germany; 4 Biopsychology, Faculty of Psychology, Institute of Cognitive Neuroscience, Ruhr University Bochum, 44801 Bochum, Germany; 5 Faculty of Medicine and University Hospital, Institute for Clinical Chemistry, University of Cologne, 50935 Cologne, Germany

**Keywords:** C-reactive protein (CRP), lymphocyte-to-monocyte ratio (LMR), neutrophil-to-lymphocyte ratio (NLR), derived NLR, platelet-to-lymphocyte ratio (PLR), melanoma, recurrence-free survival, sentinel lymph node metastasis, overall survival

## Abstract

**Simple Summary:**

Indicators of a potential recurrence of melanoma in patients after the detection of lymph node metastasis are needed in order to not treat patients unnecessarily with a systemic therapy. Blood parameters such as the number of white blood cells and ratios of different white blood cell subtypes are collected in the clinical routine and could be useful indicators of a possible disease relapse. The aim of our present study was to identify blood parameters which predict the recurrence of melanoma in melanoma patients with microscopic sentinel lymph node metastasis. We identified the lymphocyte-to-monocyte ratio (LMR) and C-reactive protein (CRP) to be the strongest predictors for melanoma recurrence.

**Abstract:**

Although adjuvant therapies with immune checkpoint inhibitors (ICI) and BRAF/MEK inhibitors improve recurrence-free survival (RFS) in stage III melanoma patients significantly, prognostic factors are needed to identify patients with a high risk of disease recurrence. Therefore, the aim of our study was to investigate the prognostic potential of routinely collected blood parameters for stage III melanoma patients with microscopic sentinel lymph node (SLN) metastasis. Altogether, we retrospectively analyzed 138 stage III melanoma patients who were diagnosed with microscopic SLN metastasis at the skin cancer center of the University Hospital Cologne between 2011 and 2020 and who did not receive prior adjuvant therapy with ICI or BRAF/MEK-inhibitors. Univariate and multivariate Cox regression analyses, Kaplan–Meier survival analyses and receiver operating characteristic (ROC) curves were performed to assess the impact of preoperatively collected blood parameters and blood ratios on recurrence-free survival (RFS; primary endpoint) and overall survival (OS). A high neutrophil-to-lymphocyte ratio (NLR), low lymphocyte-to-monocyte ratio (LMR) and high C-reactive protein (CRP) value were significantly associated with shorter RFS in multivariate analysis. For LMR (cut-off 3.5) and for CRP (cut-off 3.0) this effect remained after dichotomization. CRP showed a stronger association with RFS than NLR or LMR, with the highest association being detected for the combination of low LMR and high CRP. Additionally, derived NLR ≥ 2.0 was significantly associated with shorter OS in multivariate analysis. In summary, our data suggest that CRP in combination with LMR should be considered as a marker for melanoma recurrence in stage III melanoma patients with microscopic SLN metastasis.

## 1. Introduction

Melanoma accounts for 4.5% of all cancer diagnoses in Germany, with a rising incidence worldwide [1]. Despite a number of new therapeutic options, it remains the leading cause of death by skin cancer. At first diagnosis, melanoma is excised completely with histopathological determination of Breslow thickness and ulceration of the primary. For melanoma with a Breslow thickness ≥1 mm, a sentinel lymph node biopsy (SLNB) is recommended in addition to wide local excision. To identify the sentinel lymph node (SLN), the surgeon injects a radioactive substance, blue dye or both, at the site of the primary melanoma. After resection of the SLN, it is histopathologically assessed for the presence of lymph node metastases [2].

The presence of regional lymph node metastases is an important prognostic factor for disease progression in newly diagnosed melanoma [3,4]. For patients with loco-regionally metastasized melanoma (stage III melanoma), 5-year melanoma-specific survival ranges from 93% (stage IIIA) to 83% (stage IIIB), 69% (stage IIIC) and 32% (stage IIID) compared to 98% in stage I melanoma patients [5]. Metastases, which are only detectable by histopathological analysis, but not clinically, are defined as microscopic SLN node metastases.

Prospectively randomized trials showed improved recurrence-free survival (RFS) in stage III melanoma patients with adjuvant systemic therapy with immune checkpoint inhibitors (ICI) or BRAF/MEK targeted therapy. Still, a large number of patients do not benefit from adjuvant therapy, either because of primary or secondary resistance to adjuvant treatment or because they would not have developed metastases even without adjuvant treatment [6,7]. These patients unnecessarily endure the risks of therapy-related adverse events such as the inflammation of organ systems. Therefore, parameters which allow the identification of patients at risk for recurrence and progression are needed.

Systemic inflammation plays an important role in cancer development and progression [8]. This opens up the possibility of using specific inflammatory blood values to predict survival outcome. Amongst other cell types, neutrophils, monocytes and platelets secrete pro-tumorigenic cytokines such as tumor necrosis factor-α and vascular endothelial growth factor [9]. As the neutrophil-to-lymphocyte ratio (NLR) is a prognostic factor for overall (OS), recurrence-free (RFS) and progression-free survival (PFS) in different solid tumors [10,11], several studies investigated its role as a prognostic factor for melanoma in different tumor stages [12,13,14,15,16]. Additionally, further blood cell ratios such as the lymphocyte-to-monocyte ratio (LMR) [14,17], the platelet-to-lymphocyte ratio (PLR) [18,19] and the derived neutrophil-to-lymphocyte ratio (dNLR) [20,21], as well as serological inflammatory parameters (e.g., the acute phase reactant C-reactive protein; CRP) have been assessed for their ability to predict progression and survival in melanoma patients [22,23]. While many studies found associations between these blood parameters with disease recurrence, tumor load or survival, there are also contradictory results. Especially in localized melanoma stages, there is no consensus on which blood cell variables may add information to the established prognostic parameters (such as tumor stage) [12,15,16,21,24]. At present, it is therefore unclear which inflammatory blood parameters have the highest prognostic value, specifically in stage III melanoma patients with microscopic SLN metastasis.

The aim of our present study was to identify specific blood values (leukocyte count, neutrophil count, CRP) and blood ratios (NLR, dNLR, LMR) that correlate with RFS and OS in melanoma patients with microscopic SLN metastasis and to determine optimal cut-off values to facilitate their use in clinical routine. Moreover, we wanted to assess which of these markers have the highest prognostic potential and could help to identify patients at risk of melanoma recurrence who should be monitored more closely and receive adjuvant therapy.

## 2. Materials and Methods

### 2.1. Study Design

**Figure 1:**
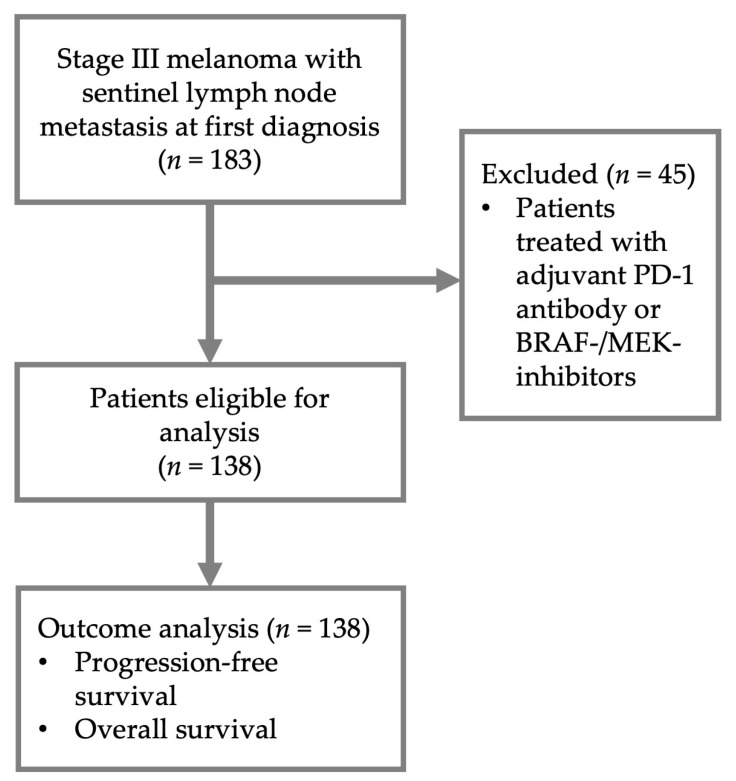
Study flow. 183 patients with stage III melanoma with microscopic sentinel lymph node metastasis were identified at the Department of Dermatology at the University Hospital Cologne. Of these, 138 patients were eligible for analysis.

Patients with cutaneous melanoma who received a SLNB with histologically, but not clinically, detectable (microscopic) lymph node metastasis between January 2011 and December 2020 at the skin cancer center of the Department of Dermatology at the University Hospital Cologne were identified from the electronic database. Of 183 patients identified, we excluded those who received adjuvant treatment with PD-1- or BRAF/MEK-inhibitors. Patients who received adjuvant interferon-α (32.6%), however, were included. This way we identified 138 melanoma patients who received a SLNB at our department and did not receive adjuvant therapy with PD-1- or BRAF/MEK-inhibitors. These were included in our final analysis (see Figure 1).

Patient and tumor characteristics (age, immunosuppressive co-medication, tumor stage, presence of an ulceration of the primary tumor, number of affected SLN and maximal size of SLN metastasis, capsule invasion by the metastasis), blood test results of blood samples drawn within 15 days before SLNB and the overall course of the disease (adjuvant treatment, recurrence, progression, survival) were collected from clinical records of the Department of Dermatology and Venereology at the University Hospital Cologne. The following blood ratios were calculated from the blood test results already provided: NLR, dNLR, LMR and PLR. These ratios were calculated from absolute blood values as follows: NLR = absolute neutrophil count/absolute lymphocyte count, dNLR = absolute neutrophil count/(absolute leukocyte count—absolute neutrophil count), LMR = absolute lymphocyte count/absolute monocyte count and PLR = absolute platelet count/absolute lymphocyte count. Laboratory analyses took place at the Institute for Clinical Chemistry at the University Hospital Cologne. The complete blood counts were performed on an automated Sysmex analyzer (Kobe, Japan) using fluorescence flow cytometry for measuring leukocytes and performing leukocyte differentiation. CRP values were measured on a Cobas c702 analyzer (Roche Diagnostics) issuing a latex-enhanced immunoturbidimetric assay. The analytical measurement range was 0.3–350.0 mg/L. The primary study endpoint was RFS and secondary endpoint was OS. The data cut point was 1 January 2022 and events after this date were censored in later survival analyses (right-censoring). This research was approved by the Ethics Committee of the University of Cologne (approval no. 20-1584).

### 2.2. Statistical Analysis

We performed univariate and multivariate Cox proportional hazards regression analyses, Kaplan–Meier survival analyses and ROC-curves to assess the impact of routinely collected blood parameters and their respective ratios on RFS and OS.

In an initial explorative analysis, we checked for intercorrelations among our clinical variables before constructing the multivariate Cox regression model. For the considered blood parameters, we conducted a manual parameter selection following a set of clinical and statistical exclusion criteria to ensure parsimony of the multivariate model and to reduce multicollinearity. These exclusion criteria were as follows: (1) blood values were excluded if they were not significant predictors of RFS or OS at an alpha level of 0.05 in univariate or multivariate Cox regression analysis; (2) blood values with missing data in >25% of the blood sample, i.e., less than 100 reported cases, were excluded to avoid a reduction in the sample size; (3) whenever absolute blood values were provided, we excluded relative blood values, as absolute counts are suggested to provide better diagnostic support [25]; (4) for blood parameters with high intercorrelations (pairwise Pearson *r* ≥ 0.7), the parameter with the weaker effect on RFS or OS was dropped, respectively. The following parameters were then included in the multivariate Cox regression analysis: age, tumor stage, presence of capsule invasion, adjuvant therapy with interferon-α, diameter of the largest SLN metastasis, as well as, respectively, one of the blood parameters selected above (NLR, dNLR, LMR, CRP; absolute leukocyte count, absolute neutrophil count).

To facilitate the clinical use of the recommended diagnostic blood parameters, we determined cut-off values by contrasting sensitivity and specificity from ROC curves for RFS and OS, respectively. For cases in which cut-off points were not immediately apparent, we evaluated the effects of different values using Kaplan–Meier estimates, as well as their effects in the univariate and multivariate Cox proportional hazards analysis.

Median follow-up time was calculated as time from first diagnosis until last patient contact or in case of death, until cut-off date. RFS was defined as time from first diagnosis until disease recurrence (locoregional or distant metastasis) or last patient contact (censored RFS) and OS as time from first diagnosis until death or last patient contact (censored OS). Differences in Kaplan–Meier estimates were assessed by two-sided log-rank test. *p*-values < 0.05 were considered statistically significant. Patients with missing data were excluded from the respective analyses. All statistical analyses were performed with IBM SPSS Statistics 27.

## 3. Results

### 3.1. Patient and Disease Characteristics

Of the 138 patients eligible for analysis, 83 patients (60%) were older than 65 years. The median age at the first diagnosis was 59 years. Seventy-one patients (51%) were male. Median follow-up time was 53.4 months (interquartile range: 28.5–70.3) after first diagnosis. According to AJCC 8th edition, 36 patients (26%) had stage IIIA melanoma at first diagnosis, 32 (23%) stage IIIB and 70 patients (51%) stage IIIC. No cases with stage IIID were recorded. Forty-five patients (33%) received adjuvant interferon-α therapy. Median Breslow thickness was 3.05 mm and ranged from 0.82 to 16.00 mm. Sixty-four primary melanomas (46%) were ulcerated and ninety-five (69%) had a mitosis rate > 1/mm^2^. The number of SLN metastases ranged from one lymph node metastasis (108 patients, 78%) to two (22 patients, 16%) and up to three lymph node metastases (8 patients, 6%), with the median size of the biggest metastasis being 1.25 mm and 11% showing capsule invasion. Median RFS time was 1.9 years, with a minimum time of 51 days and a maximum of 9.6 years. Seventy-six patients (55%) showed tumor progression within the study period. Of these, sixty-one (80%) developed distant metastases and thereby showed a stage shift from stage III to IV. For OS, the median survival time was 4.5 years with a minimum of 51 days and a maximum recorded time of 9.9 years. Forty-three patients (31%) died during the observed study period. For an overview of patient and survival characteristics see Table 1; for further disease characteristics, see Table S1.

### 3.2. Exploratory Analysis

**Figure 2:**
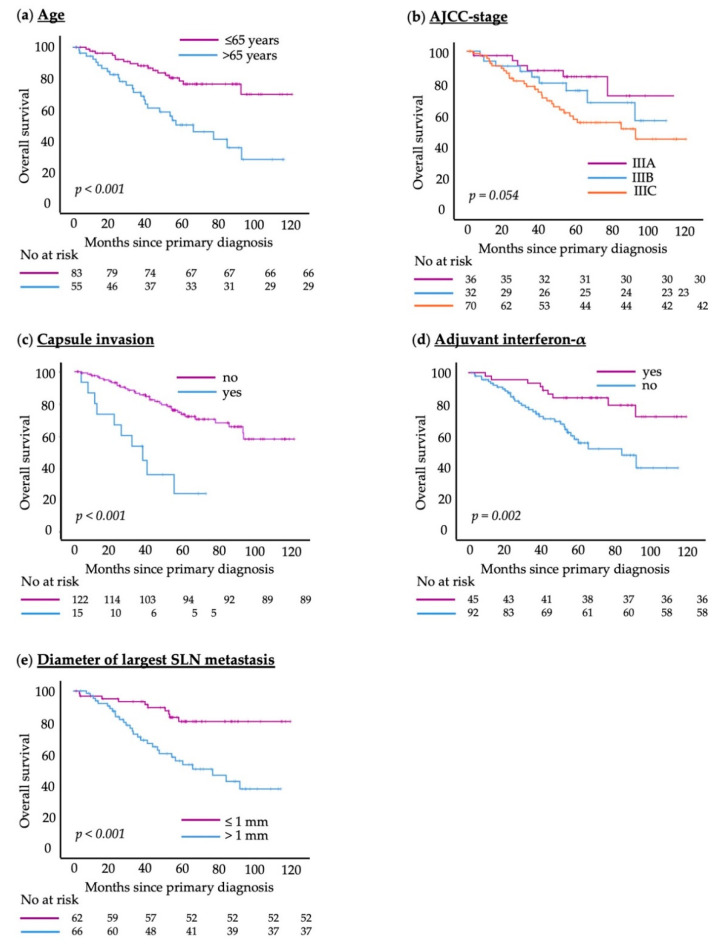
Kaplan–Meier survival curves showing recurrence-free survival for covariates of the multivariate Cox regression model: (**a**) patient age; (**b**) AJCC-stage; (**c**) capsule invasion of SLN metastasis; (**d**) adjuvant interferon-α therapy; (**e**) diameter of largest SLN metastasis. The log-rank test was used to compare between groups; *p* < 0.05 was considered significant.

Univariate Cox proportional hazards analyses revealed multiple parameters that were significantly associated with shorter RFS: age > 65 years (HR = 1.985, 95%CI = 1.261–3.125, *p* = 0.003), largest diameter of the SLN metastasis > 1 mm (HR = 1.849, 95%CI = 1.139–3.004, *p* = 0.013), the presence of an ulceration in the primary tumor (HR = 2.390, 95%CI = 1.505–3.794, *p* < 0.001) and AJCC-stage IIIC versus stage IIIA (HR = 4.303, 95%CI = 2.114–8.759, *p* < 0.001). There was no significant difference for AJCC-stage IIIB compared to stage IIIA. Patients who received a CLND had a higher risk of recurrence in the univariate analysis (HR = 1.710, 95%CI = 1.072–2.729, *p* = 0.024). Figure 2 (RFS) and Figure S1 (OS) show Kaplan–Meier survival curves of some important variables.

Similarly, in the univariate analysis, the following parameters were associated with shorter OS: age > 65 years (HR = 3.060, 95%CI = 1.657–5.650, *p* < 0.001), higher AJCC-stage (HR = 2.685, 95%CI = 1.111–6.490, *p* = 0.028; specifically, stage IIIC versus stage IIIA), largest diameter of the SLN metastasis > 1 mm (HR = 3.444, 95%CI = 1.674–7.086, *p* < 0.001) and the presence of ulceration of the primary tumor (HR = 2.044, 95%CI = 1.108–3.770, *p* = 0.022). Capsule invasion of the SLN by the metastasis (HR = 4.394, 95%CI = 2.120–9.109, *p* < 0.001) was also associated with significantly decreased OS. Patients who received adjuvant interferon-α therapy (HR = 0.329, 95%CI = 0.156–0.692, *p* = 0.003) had a significantly lowered risk of death and therefore a better OS in the univariate analysis.

Gender, type of melanoma, number of affected lymph nodes in SLNB, mitosis rate (>1/mm^2^ versus ≤1/mm^2^) and presence of locoregional cutaneous metastasis showed no significant association with any of the defined endpoints in the univariate analysis (Table S3).

### 3.3. Pre-Selection of Relevant Blood Parameters

**Figure 3:**
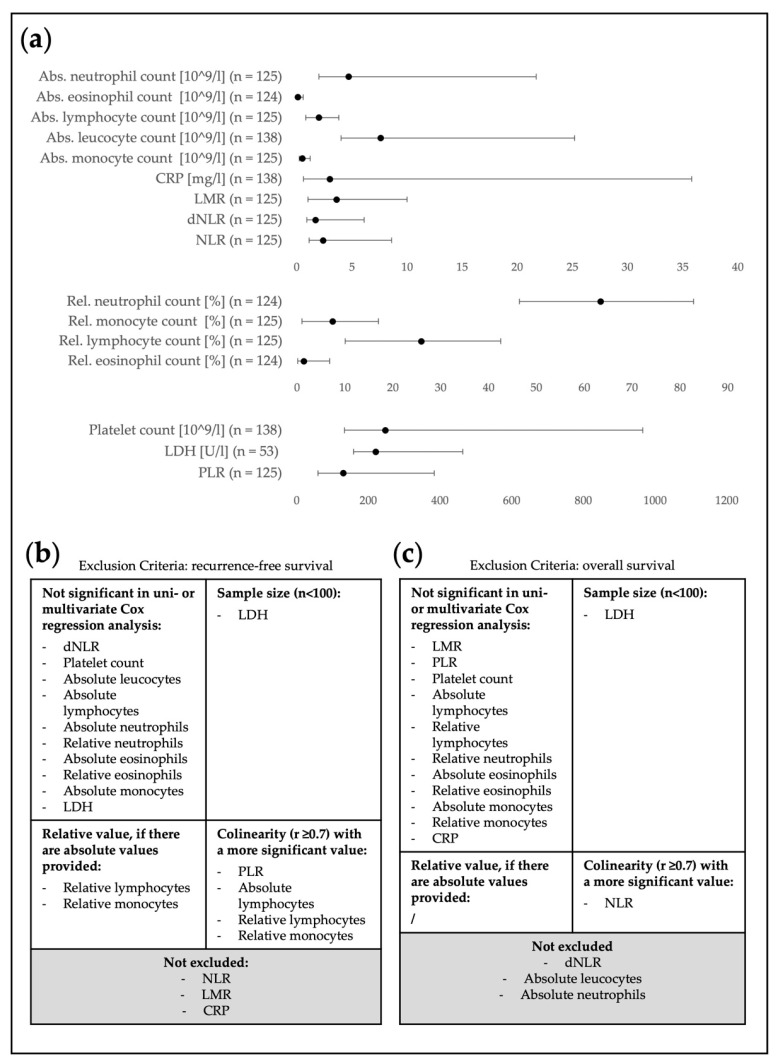
Overview of all blood values with applied exclusion criteria. Panel (**a**) shows a forest plot distribution of median values with 95%CI on three different scales. Panel (**b**) shows a list of excluded blood parameters related to recurrence-free survival and panel (**c**) shows excluded parameters for overall survival. CRP: C-reactive protein, LMR: lymphocyte-to-monocyte ratio, NLR: neutrophil-to-lymphocyte ratio, dNLR: derived NLR, LDH: lactate dehydrogenase, PLR: platelet-to-lymphocyte ratio.

After a manual parameter selection following the criteria outlined above (direct effect on endpoints, patient number, feature simplicity and multicollinearity), three blood parameters out of sixteen were selected as relevant predictors of recurrence (see Figure 3 and Table S2a for details). NLR, LMR and CRP were significantly associated with RFS in the univariate Cox regression analysis (NLR: HR = 1.236, 95%CI = 1.064–1.437, *p* = 0.006; LMR: HR = 0.689, 95%CI = 0.564–0.841, *p* < 0.001; CRP: HR = 1.065, 95%CI = 1.026–1.105, *p* < 0.001, respectively). These parameters were available for ≥100 patients, were calculated based on absolute blood values, or were absolute values themselves and were clinically and statistically more relevant than other intercorrelated values.

The same exclusion criteria revealed three different blood parameters to be particularly relevant for the prediction of OS: dNLR, absolute leukocyte count and absolute neutrophil count. Of note, the selected parameters were not significantly associated with OS in the univariate analysis, but had a significant independent effect when they were controlled for age, AJCC-stage, capsule invasion, adjuvant interferon-α therapy and diameter of the largest metastasis in the multivariate analysis (dNLR: HR = 1.410, 95%CI = 1.024–1.942, *p* = 0.035; absolute leukocyte count: HR = 1.334, 95%CI = 1.022–1.742, *p* = 0.034; absolute neutrophil count: HR = 1.404, 95%CI = 1.086–1.815, *p* = 0.010, see Figure 3 and Table S2b).

### 3.4. Cut-off Points for Blood Values

For the set of selected blood parameters listed above (NLR, LMR and CRP for RFS; dNLR, absolute leukocyte count and absolute neutrophil count for OS), we determined cut-off values by contrasting sensitivity and specificity from ROC curves, evaluated the effects of different alternative values using Kaplan–Meier estimates and analyzed their effects in the univariate and multivariate Cox proportional hazards analysis. This enabled dichotomization (separation of the respective parameters into two groups: above and below the cut-off point). These cut-off points of the selected blood parameters were used in later analyses and considerably simplify the clinical applications of these parameters.

**Figure 4:**
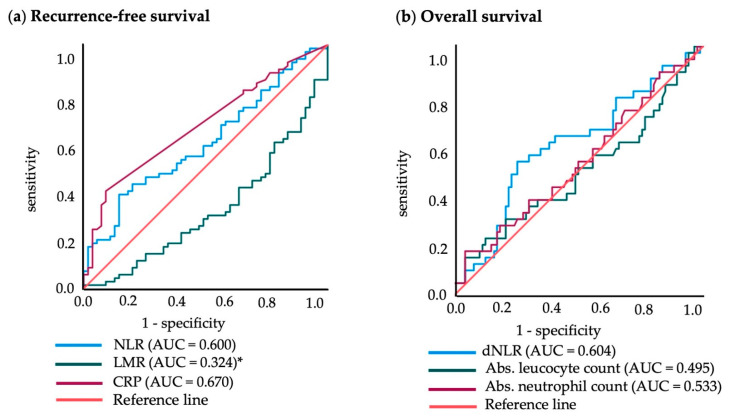
ROC curves for recurrence-free survival (RFS) and overall survival (OS) for dichotomization of blood values: (**a**) sensitivity and specificity with neutrophil-to-lymphocyte ratio (NLR); lymphocyte-to-monocyte ratio (LMR); C-reactive protein (CRP) for RFS survival; (**b**) sensitivity and specificity with derived NLR (dNLR), absolute leukocyte and neutrophil count for OS. (* Note: since the LMR negatively correlates to RFS, the ROC curve is below the reference line).

**Figure 5:**
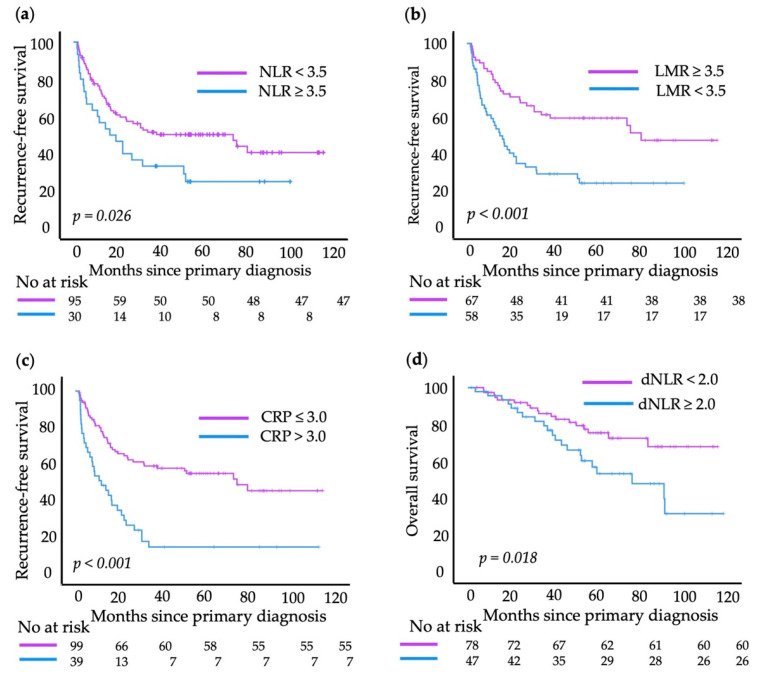
Kaplan–Meier survival curves showing recurrence-free survival (RFS) and overall survival (OS) for dichotomized blood values: (**a**) neutrophil-to-lymphocyte ratio (NLR); (**b**) lymphocyte-to-monocyte ratio (LMR); (**c**) C-reactive protein (CRP); (**d**) derived NLR (dNLR). The log-rank test was used to compare between groups; *p* < 0.05 was considered significant.

Figure 4 shows the ROC curves of these parameters for RFS and OS, respectively. For NLR, the optimal cut-off point was rounded to 3.5 (sensitivity 0.314; 1-specificity 0.145). The LMR showed the highest significance for RFS prediction at a cut-off point of 3.5 (sensitivity 0.414; 1-specificity 0.691). A clear cut-off for CRP could not be identified in the ROC analysis (range 3.0–5.0). It was set to 3.0 (sensitivity 0.400; 1-specificity 0.091), as thereby the two groups were most balanced in size. For OS, the optimal cut-off point for the dNLR was 2.0 (sensitivity 0.513; 1-specificity 0.256). For the absolute leukocyte count and absolute neutrophil count no cut-off values could be identified, as the area under the curve (AUC) was approximately 0.5 for both parameters, which indicates a low predictive value. Log rank analysis confirmed the significance of the determined cut-off values for RFS with *p* = 0.026 for NLR, *p* < 0.001 for LMR and *p* < 0.001 for CRP. For OS, only the dichotomized dNLR showed statistically significant results (*p* = 0.01; Figure 5).

### 3.5. Hierarchical Multivariate Cox Regression Analysis

With the parameters pre-selected and investigated above, we proceeded to build a multivariate Cox regression analysis model with hierarchically organized control variables. First, we controlled for the patients’ age. Gender showed no relevance in our previous exploratory analysis (see Table S3). Next, from already described prognostic factors in literature and medical guidelines, we chose the AJCC-stage, which was significantly associated with RFS and OS (see Table S3) and indirectly includes many strong prognostic variables (such as the presence of ulceration of the primary, Breslow thickness, number of affected lymph nodes, presence of cutaneous metastases). In addition, capsule invasion of the lymph node by the metastasis and whether patients received adjuvant therapy with interferon-α were included as variables. Subsequently, our pre-selected blood values were included individually into the model to determine their respective prognostic value, with respect to the two endpoints. Table 2 and Table 3 show the results of the multivariate Cox regression analyses for RFS and OS, while Table 4 shows an overview of the univariate and multivariate Cox regression analyses with continuous and dichotomized blood values described in the previous section.

As shown in Table 2, a low LMR-value (<3.5) and a high CRP-value (>3.0) were independently associated with an increased risk of progression in the multivariate analysis (LMR: HR = 2.198, 95%CI = 1.301–3.715, *p* = 0.003; CRP: HR = 3.355, 95%CI = 2.017–5.582, *p* < 0.001). Note that the NLR was independently associated with RFS as a continuous factor (HR = 1.340, 95%CI = 1.050–1.711, *p* = 0.019), but not as dichotomized parameter with a cut-off at 3.5 (HR = 1.512, 95%CI = 0.845–2.705, *p* = 0.164; see Table 4). Comparing the effect sizes of CRP and LMR above, CRP had a higher effect on RFS than LMR (CRP > 3.0 associated with a 3.4-fold increased risk of progression compared to LMR ≥ 3.5 with a 2.2-fold increased risk of progression). Furthermore, in the multivariate Cox regression analysis for RFS with CRP as the dichotomized value (see Table 2), AJCC-stage (IIIC versus IIIA: HR = 3.707, 95%CI = 1.784–7.703, *p* < 0.001) and age at first diagnosis (HR = 1.402, 95%CI = 1.054–1.865, *p* = 0.020) were also independently associated with RFS. Capsule invasion, the maximum diameter of the biggest SLN metastasis and adjuvant treatment with interferon-α were not independently associated with RFS in our model.

In the multivariate Cox regression model for OS (Table 3) with dNLR as the dichotomized value with a cut-off at 2.0, a higher dNLR was significantly associated with a shorter OS (HR = 2.428, 95%CI = 1.186–4.968, *p* = 0.015). In this model, the maximum diameter of the biggest SLN metastasis was also an independent significant prognostic factor (HR = 1.272, 95%CI = 1.071–1.511, *p* = 0.006). Age, AJCC-stage, capsule invasion and adjuvant therapy with interferon-α lost the previously found univariate effect on OS after controlling for dNLR (see Figure S1). The NLR was also significantly associated with OS in the multivariate Cox regression analysis. As this effect was smaller than the one of dNLR, we further analyzed dNLR.

### 3.6. Hierarchical Multivariate Cox Regression Analysis

Next, the power of the final multivariate Cox regression models was tested in a ROC curve analysis. A model for RFS with the defined covariates, in addition to one of our analyzed blood values, showed the following total area under the curve (AUC): NLR 0.748; LMR 0.772 and CRP 0.801. In comparison, the models without the additional blood parameters showed a baseline AUC of 0.744. For OS, the area under the curve for the model with dNLR was higher (AUC = 0.767) than the baseline model without dNLR (AUC = 0.733).

### 3.7. Combination of Dichotomized Blood Values

We created new variables denoting samples with NLR ≥ 3.5 plus CRP > 3.0, LMR < 3.5 plus CRP > 3.0, NLR ≥ 3.5 plus LMR < 3.5 and NLR ≥ 3.5 plus LMR < 3.5 plus CRP > 3.0, to assess whether these combinations were associated with a bigger prognostic effect than the single blood values. The combination of high NLR and high CRP shows a HR = 4.838 (95%CI = 2.009–11.652, *p* < 0.001), compared to no elevation of these blood values. The further addition of low LMR to high NLR and high CRP led to a significantly better prognostic effect on RFS (HR = 7.690, 95%CI = 2.789–21.202, *p* < 0.001), although the highest prognostic value for RFS was seen in patients with a combination of low LMR and high CRP compared to patients with LMR ≥ 3.5 and CRP < 3.0 (HR = 7.700, 95%CI = 3.436–17.255, *p* < 0.001). The prognostic effect of these combined blood values was stronger than the effects of the single blood cell ratios (LMR < 3.5: HR = 2.198 95%CI = 1.301–3.715, *p* = 0.003; CRP > 3.0: HR = 3.355, 95%CI = 2.017–5.582, *p* < 0.001; see Table 2 and Table S4 for a comparison of effect sizes).

## 4. Discussion

In our present study, we analyzed the effectiveness of different blood variables to predict RFS and OS in stage III melanoma patients with microscopically detectable SLN metastasis. To the best of our knowledge, this is the first study to comparatively assess the prognostic value of ratios of different blood cells and CRP, specifically in stage III melanoma patients with microscopic SLN metastasis. This specific setting was chosen to identify patients with a higher risk of disease recurrence from the large number of patients who are regularly diagnosed with SLN metastasis, and are potential candidates for adjuvant therapy with immune checkpoint inhibitors or BRAF/MEK-inhibitors. We therefore intentionally excluded patients who received these adjuvant therapies. Our findings demonstrate that NLR, LMR and CRP provide useful prognostic information about RFS, in addition to the predictiveness of other well-established prognostic factors. Nevertheless, the factor with the strongest association with RFS was high CRP, with the combination of high CRP and low LMR having the strongest predictive power for melanoma recurrence in our patient cohort. Regarding OS, a high dNLR and NLR were associated with shorter OS.

The NLR contains information about the number of lymphocytes, which are known for their strong anti-tumoral effects [26], and about the number of neutrophils, which are often reported to be pro-tumorigenic [15]. While neutrophils in the later tumor stages are mainly pro-tumorigenic, they can also exert anti-tumoral effects. These are most often reported in early tumor stages [15,27,28]. An increased NLR has been regarded to mirror sustained angiogenesis and proliferation of tumor cells [29].

Although there are numerous studies on the prognostic value of NLR in advanced melanoma and on the predictive potential of NLR in the treatment with ICI or BRAF/MEK-inhibitors, few studies analyze the prognostic potential of NLR and other blood ratios in localized melanoma stages, especially in patients who did not receive adjuvant ICI or targeted therapy. For example, a retrospective study by Ma et al. investigated patients with stage III melanoma and described an NLR ≥ 2.5 to be a strong predictor for disease recurrence [30]. In our study, NLR as a continuous variable was also associated with higher risk of progression and shorter RFS. In univariate analysis, this effect also remained for the dichotomized value of NLR (cut-off at 3.5), but after adjustment for confounding factors in the multivariate analysis, the dichotomized value did not show a significant association with RFS. These differences could be explained by differences in the patient cohort. While both studies investigated stage III melanoma patients, we excluded patients who received adjuvant therapy with ICI or targeted therapy. Furthermore, we only included patients with microscopic SLN metastasis and therefore excluded stage III melanoma patients with macroscopic metastasis or only cutaneous metastases, who are known to have an inferior prognosis.

Lino-Silva et al. were the first to analyze NLR in stage I-III patients with localized melanoma. They described an NLR ≥ 2.0 as a prognostic marker for shorter OS, although in subgroup analysis this only remained significant for stage II patients. Their group also found an association of NLR ≥ 2.0 with lymph node metastasis and recurrence [16]. We found high continuous NLR values in our multivariate analysis to be significantly associated with shorter RFS and OS in our study population. Furthermore, we found a high dNLR (not assessed by Lino-Silva et al.) to have an even more significant and stronger association with a shorter OS than a high NLR.

The largest study that assessed the prognostic value of the baseline NLR in stage I-III melanoma patients was performed by Robinson et al. They included 1077 patients with negative SLN, 274 with microscopic and 138 with macroscopic SLN metastasis. The NLR increased with tumor stage and was lowest in patients without lymph node metastasis, higher in patients with microscopic lymph node metastasis and highest in those with macroscopic metastasis. They did not find a correlation between NLR and recurrence, but found an association between the tumor burden at first diagnosis and NLR [13]. In our study, NLR was an independent prognostic factor for RFS and OS in the multivariate analysis, which included the diameter of the SLN metastasis (marker for tumor burden).

Contrary to most studies and to our results, Wade et al. reported a low baseline NLR and PLR to be associated with shorter OS and MSS in patients with stage I-III melanoma. Since this association was only detectable in multivariate analysis, but not in univariate analysis, they suggested that in low tumor stages the elimination of confounding factors is even more important. Interestingly, we also experienced that NLR and dNLR only showed a significant effect on OS after adjusting for confounding factors. As opposed to the study by Wade et al., in our stage III cohort with microscopic SLN metastasis, a high NLR or high dNLR predicted a shorter OS. A possible reason for Wade et al.’s opposing results compared to ours, and those of other studies investigating melanoma with stage III disease, could be the high number of patients with stage I melanoma (890 patients compared to 184 patients with stage II and 274 patients with stage III melanoma) they included [15].

Recently, several studies were published analyzing the predictive value of the dNLR. These were mainly performed in advanced melanoma patients (stage IV and not operable stage III). Ferrucci et al. and Capone et al. retrospectively analyzed advanced melanoma patients who were treated with ICI (ipilimumab or nivolumab). Ferrucci and colleagues found a high dNLR and absolute neutrophil count to be independently associated with an increased risk of death and disease progression. Capone et al. described an NLR > 5.0 and dNLR > 3.0 at baseline to be associated with shorter OS and shorter progression-free survival (PFS) [20,21]. Our study supports these findings, although we used different cut-off values and analyzed patients with resectable stage III melanoma. We identified a dNLR ≥ 2.0 to be the strongest and most significant independent predictor for shorter OS. Interestingly, the dNLR was not associated with RFS even though studies have shown similar prognostic values for NLR and dNLR in cancer patients, suggesting that the lymphocyte fraction is accurately estimated by the result of the absolute neutrophil count subtracted from the absolute leukocyte count in the denominator of dNLR [31]. The dNLR is, however, not exclusively determined by neutrophil and lymphocyte values but also by all other white blood count subtypes in the denominator, with monocytes accounting for the other main fraction. As these have been reported to be lower in patients with malignancies [14,17,32,33], they might have led to an inaccurate dNLR calculation.

While several retrospective studies in stage IV melanoma patients showed a significant association of the LMR with OS [17,32,33], there are few reports about the role of the LMR in localized cutaneous melanoma so far. In our study, a low LMR was an independent prognostic marker for shorter RFS. Similarly, Wang et al. identified an LMR ≤ 7.38 and surgery as factors significantly associated with worse PFS and OS in a univariate analysis in patients with mucosal melanoma. In their multivariate analysis, significance was only maintained for OS [14]. In our study, there was no significant association between LMR and OS. Interestingly, and opposed to these findings, Wade et al. revealed an association of a low LMR (and high NLR) with a regression of the primary melanoma for stage I-III patients [15]. In addition, Gandini et al. found patients with distant metastases to have higher absolute leukocyte, neutrophil and monocyte counts and lower lymphocyte counts in comparison to stage I-III patients. There was no association of peripheral blood cell counts with OS in localized or regionally metastasized melanoma patients. Only the blood values of stage IV patients with distant metastasis showed an association with OS. They suggest that a high neutrophil, low monocyte and low lymphocyte count are stronger predictors for shorter OS in advanced tumor stages compared to earlier tumor stages [34]. While we detected an association between a high relative lymphocyte count and low LMR with RFS, we did not find any association between a high monocyte or lymphocyte count or LMR with OS.

We found CRP to be the strongest independent prognostic parameter for RFS in our studied cohort. Few studies have been published on CRP as a prognostic marker for progression in melanoma patients. Fang et al. showed that CRP (as continuous baseline variable and as dichotomized value <10 mg/L, ≥10 mg/L) was associated with shorter OS and MSS in any melanoma stage. In a stage III/VI subgroup analysis, this association remained. CRP >10 mg/L in stage I/II patients was also associated with progression. All these findings remained significant after adjustment for confounding factors [22]. As opposed to Fang et al., we found a CRP baseline level of >3.0 mg/L to be a strong predictor for shorter RFS, but not for shorter OS. Most likely, these differences occurred as Fang et al. analyzed stage III and stage IV patients together in one subgroup, while we focused on a specific stage III melanoma cohort. In published studies so far, the defined cut-off value for CRP was often 10 mg/L. Compared to these, our cut-off value of 3.0 mg/L is rather low. Possibly, the lower cut-off value emerged because the performing laboratory in our study routinely reports quantitative CRP levels down to the lower detection limit of 0.3 mg/L. CRP values originating from other laboratories are sometimes simply reported as being below the detection limit for inflammation, which is <5 mg/L. Since our determined optimal cut-off value of 3.0 mg/L is below this detection limit, we suggest quantifying CRP also in the low range beneath 5 mg/L.

In addition to the independent assessment of the predictive value of single blood parameters, we assessed CRP, LMR and NLR in different combinations for their ability to predict disease progression. Doing so, we detected that a combined score of high CRP and low LMR was the strongest and most significant prognostic factor for melanoma recurrence when compared to all investigated parameters.

A limitation of this study is its retrospective nature and that it was conducted as a single-center study. To better assess the effects of the analyzed parameters on OS, a larger patient cohort with more death events would have been needed. Nevertheless, the strictly uniform patient cohort (only stage III patients with microscopic disease) and the fact that all important known prognostic parameters were included in this study are a strength compared to other studies which included patients from stage I–III or stage III patients with microscopic and macroscopic disease, as well as patients with only cutaneous metastases. In addition, RFS is an accepted surrogate marker for OS and is the most commonly used primary endpoint in prospective randomized adjuvant trials with melanoma patients. While not being able to exclude patients who received interferon-α as adjuvant therapy, we excluded patients who received adjuvant PD-1-inhibitors or BRAF/MEK-inhibitor therapy, which (unlike interferon-α) have been shown to have a strong effect on RFS in randomized phase III trials. In addition, to account for possible effects of interferon-α, we included treatment with interferon-α as a control variable in our multivariate analysis.

In summary, although a number of studies assess the prognostic value of different blood parameters such as NLR in melanoma patients, this study is the first to systematically comparatively analyze the prognostic value of different ratios of blood parameters and CRP in melanoma patients with microscopic SLN metastasis. This uniform patient cohort is a strength of this study since different tumor stages may confound the results in other studies that assessed some, but not all of these parameters. Our analysis shows that the prognostic value of CRP for RFS is much higher in this patient cohort than that of the widely discussed NLR.

## 5. Conclusions

Altogether, we detected strong associations of NLR, LMR and CRP with RFS, which were independent of other known prognostic parameters in our cohort of stage III melanoma patients with microscopic SLN metastasis. CRP was the parameter with the strongest association with RFS compared to the other parameters. Nevertheless, the combination of CRP and LMR was associated with the strongest potential to predict progression. Therefore, we propose to consider these variables when assessing a patient’s risk of disease recurrence, the necessity for closer monitoring, or for an adjuvant therapy. Nevertheless, further studies in larger, prospectively collected patient cohorts are required to validate our findings.

## Figures and Tables

**Table 1:** Baseline characteristics and patient outcome.

Patient Characteristics and Outcome	All Patients
	***n* = 138 (100%)**
**Age**	
≤65 years	83 (60.1)
>65 years	55 (39.9)
Median in years (IQR) *	59 (45.0–72.0)
**Gender**	
Male	71 (51,4)
Female	67 (48.6)
**Adjuvant interferon-α**	
No	92 (66.7)
Yes	45 (32.6)
Unknown	1 (0.7)
**AJCC-stage**	
IIIA	36 (26.1)
IIIB	32 (23.2)
IIIC	70 (50.7)
IIID	/
**Recurrence (locoregional and/or distant)**	
No	62 (44.9)
Yes	76 (55.1)
-only locoregional	15 (19.7)
-locoregional and distant	61 (80.2)
**Death**	
No	95 (68.8)
Yes	43 (31.2)
**Recurrence-free survival**	
Median in months (IQR)	23.3 (8.0–56.9)
Overall survival **	
Median in months (IQR)	53.4 (28.5–70.3)

* IQR = Interquartile range (Q1–Q3). ** Censored values: 95 patients lived longer than 10 years.

**Table 2:** Multivariate Cox regression analysis for recurrence-free survival (RFS) either with C-reactive protein (CRP), lymphocyte-to-monocyte ratio (LMR) or neutrophil-to-lymphocyte ratio (NLR).

	Model 1: RFS		Model 2: RFS		Model 3: RFS	
**Variable** (reference bold)	**HR (95%CI)**	***p*-Value**	**HR (95%CI)**	***p*-Value**	**HR (95%CI)**	***p*-Value**
Age						
(continuous)	1.330 (0.974–1.817)	0.073	1.286 (0.943–1.752)	0.111	**1.402 (1.054–1.865)**	**0.020**
AJCC-stage						
(A: *n* = 35)						
(B: *n* = 28)						
(C: *n* = 63)						
IIIB vs. **IIIA**	2.143 (0.885–5.188)	0.091	2.151 (0.890–5.197)	0.089	2.077 (0.888–4.857)	0.092
IIIC vs. **IIIA**	**3.461 (1.599–7.489)**	**0.005**	**3.410 (1.575–7.384)**	**0.002**	**3.707 (1.784–7.703)**	**<0.001**
IIID vs. **IIIA**	/	/	/	/	/	/
Capsule invasion						
(*n* = 14; *n* = 112)						
(no vs. **yes**)	0.798 (0.398–1.356)	0.568	0.808 (0.371–1.762)	0.593	1.027 (0.485–2.176)	0.944
Adjuvant interferon-α						
(*n* = 84; *n* = 42)						
(yes vs. **no**)	0.735 (0.398–1.356)	0.324	0.763 (0.412–1.412)	0.389	0.779 (0.434–1.399)	0.403
Size of largest SLN metastasis						
(continuous)	1.053 (0.898–1.234)	0.524	1.028 (0.876–1.207)	0.734	1.129 (0.974–1.307)	0.107
NLR						
(*n* = 27; *n* = 87)						
(≥3.5 vs. **<3.5**)	1.512 (0.845–2.705)	0.164				
LMR						
(*n* = 51; *n* = 63)						
(<3.5 vs. **≥3.5**)			**2.198 (1.301–3.715)**	**0.003**		
CRP						
(*n* = 92; *n* = 34)						
(>3.0 vs. **≤3.0**)					**3.355 (2.017–5.582)**	**<0.001**

Note: values in bold indicate significant results.

**Table 3:** Multivariate Cox regression analysis for overall survival with derived NLR (dNLR).

	Model 4: OS	
**Variable** (Reference bold)	**HR (95%CI)**	***p*-Value**
Age		
(continuous)	1.438 (0.935–2.209)	0.098
AJCC-stage		
(*n* = 35, *n* = 28, *n* = 63, *n* = 0)		
IIIB vs. **IIIA**	2.311 (0.699–7.647)	0.170
IIIC vs. **IIIA**	2.199 (0.730–6.624)	0.161
IIID vs. **IIIA**	/	/
Capsule invasion		
(*n* = 14; *n* = 112)		
(no vs. **yes**)	0.482 (0.196–1.1885)	0.113
Adjuvant interferon-α		
(*n* = 84; *n* = 42)		
(yes vs. **no**)	0.404 (0.161–1.014)	0.053
Size of biggest SLN metastasis (continuous)	**1.272 (1.071–1.511)**	**0.006**
dNLR		
(*n* = 70; *n* = 44)		
(≥2.0 vs. **<2.0**)	**2.428 (1.186–4.968)**	**0.015**

Note: values in bold indicate significant results.

**Table 4:** Significance of neutrophil-to-lymphocyte ratio (NLR), lymphocyte-to-monocyte ratio (LMR) and C-reactive protein (CRP) for recurrence-free survival (RFS) and derived NLR (dNLR) for overall survival (OS) as continuous and dichotomized variables in univariate and multivariate Cox regression analysis.

Blood Values	Univariate Cox Regression Analysis *n* HR (95%CI) *p*-Value		Multivariate Cox Regression Analysis * n HR (95%CI) *p*-Value	
**Outcome: RFS**				
Model 1: NLR	continuous (*n* = 125)	**1.236 (1.064–1.437) 0.006**	continuous (*n* = 114)	**1.340 (1.050–1.711) 0.019**
	cut-off 3.5 (*n* = 30; *n* = 95) (≥3.5 vs. <3.5)	**1.761 (1.063–2.919) 0.028**	cut-off 3.5 (*n* = 27; *n* = 87) (≥3.5 vs. **<3.5**)	1.512 (0.845–2.705) 0.164
Model 2: LMR	continuous (*n* = 125)	**0.689 (0.564–0.841) <0.001**	continuous (*n* = 114)	**0.608 (0.422–0.877) 0.008**
	cut-off 3.5 (*n* = 58; *n* = 67) (<3.5 vs. ≥3.5)	**2.433 (1.505–3.934) <0.001**	cut-off 3.5 (*n* = 51; *n* = 63) (<3.5 vs. **≥3.5**)	**2.198 (1.301–3.715) 0.003**
Model 3: CRP	continuous (*n* = 138)	**1.065 (1.026–1.105) <0.001**	continuous (*n* = 126)	**1.457 (1.214–1.747) <0.001**
	cut-off 3.0 (*n* = 39 vs. 99) (>3.0 vs. ≤3.0)	**2.841 (1.791–4.508) <0.001**	cut-off 3.0 (*n* = 34 vs. 92) (>3.0 vs. **≤3.0**)	**3.355 (2.017–5.582) <0.001**
**Outcome: OS**				
Model 4: dNLR	continuous (*n* = 124)	1.287 (0.945–1.753) 0.109	continuous (*n* = 114)	**1.410 (1.024–1.942) 0.035**
	cut-off 2.0 (*n* = 47; *n* = 78) (≥2.0 vs. <2.0)	**2.102 (1.119–3.948) 0.021**	cut-off 2.0 (*n* = 44; *n* = 70) (≥2.0 vs. **<2.0**)	**2.428 (1.186–4.968) 0.015**

* Following parameters were additionally included in the multivariate Cox regression analysis: age, AJCC-stage, capsule invasion, adjuvant interferon-α, size of largest SLN metastasis. Note: values in bold indicate significant results.

## Data Availability

All relevant data from this study can be found in the manuscript or in the provided supplementary material.

## Supplementary Materials

The following supporting information can be downloaded at: https://www.mdpi.com/article/10.3390/cancers15030702/s1, Table S1a: Baseline tumor characteristics (primary; *n* = 138); Table S1b: Baseline tumor characteristics (lymph node metastasis; *n* = 138); Table S2a: Overview of results of all blood variables in univariate and multivariate Cox regression analysis for recurrence-free survival (RFS) with applied exclusion criteria; Table S2b: Overview of results of all blood variables in univariate and multivariate Cox regression analysis for overall survival (OS) with applied exclusion criteria; Table S3: Univariate Cox regression analysis with covariates for recurrence-free survival and overall survival; Table S4: Univariate and multivariate Cox regression analysis with combined blood values of NLR, LMR and CRP for recurrence-free survival; Figure S1: Kaplan-Meier survival curves showing overall survival for covariates of the multivariate Cox regression model.
