# Supplementary material for: C-Reactive Protein and Lymphocyte-to-Monocyte Ratio Predict Recurrence in Stage III Melanoma Patients with Microscopic Sentinel Lymph Node Metastasis
Source: Cancers (Basel). 2023 Jan 23;15(3):702. doi: 10.3390/cancers15030702 (PMC9913855; doi:10.3390/cancers15030702)
Supplement: Supplementary file 1 [file cancers-15-00702-s001.zip › cancers-2124412-supplementary.pdf]

cancers

MDPI

Supplementary Materials

C-Reactive Protein and Lymphocyte-to-Monocyte Ratio Predict Recurrence in Stage III Melanoma Patients with Microscopic Sentinel Lymph Node Metastasis

Table S1a: Baseline tumor characteristics (primary; n=138).

	**All patients** n=138 (100%)
**Site of primary**	
Head and neck	4 (2.9)
Trunk	51 (37.0)
Upper extremity	20 (14.5)
Lower extremity	63 (45.7)
**Type of primary**	
SSM	39 (28.3)
NMM	63 (45.7)
ALM	6 (4.3)
LMM	0 (0.0)
other	24 (17.4)
unknown	6 (4.3)
**Tumor thickness (Breslow)**	
<1mm	3 (2.2)
≥1<2mm	36 (26.1)
≥2<4mm	53 (38.4)
≥4mm	46 (33.3)
Median in mm (IQR)*	3.1 (1.6-4.5)
**Presence of ulceration**	
No	74 (53.6)
Yes	64 (46.4)
**Mitosis rate > 1/mm^2**	
No	5 (3.6)
Yes	95 (68.8)
Unknown	38 (27.5)
*IQR = Interquartile range (Q1 – Q3).	

Table S1b: Baseline tumor characteristics (lymph; node metastasis; n=138).

	**All patients** n=138 (100%)
**Average resected number of SLN**	
1	69 (50.0)
2	38 (27.5)
3	15 (10.9)
≥4	16 (11.5)
**Number of affected SLN**	
1	108 (78.3)
2	22 (15.9)
3	8 (5.8)
**Largest diameter of SLN metastasis (in mm)**	
<1 (0.09-0.99)	
≥1 <2 (1.00-1.99)	41 (29.7)
≥2 <3 (2.00-2.99)	28 (20.3)
≥3 <4 (3.00-3.99)	19 (13.8)
≥4	14 (10.1)
Unknown	26 (18.8)
Median in mm (IQR)*	10 (7.2)
	1.3 (0.5-3.0)
**Capsule invasion of SLN metastasis**	
No	122 (88.4)
Yes	15 (10.9)
Unknown	1 (0.7)
**Presence of satellite metastasis**	
No	130 (94.2)
Yes	8 (5.8)
**CLND**	
No	66 (47.8)
Yes	72 (52.2)
-no additional metastasis in CLND	58 (61.7)
-additional metastasis in CLND	36 (38.3)

*IQR = Interquartile range (Q1 – Q3).

Table S2a: Overview of results of all blood variables in univariate and multivariate Cox regression analysis for recurrence-free survival (RFS) with applied exclusion criteria.

**Blood values** (continuous parameters)	**Univariate Cox regression analysis** n	HR (95% CI) p-value	**Multivariate Cox regression analysis*** n	HR (95% CI) p-value	**Exclusion criteria**
NLR	125	**1.236 (1.064-1.437) 0.006**	114	**1.340 (1.050-1.711) 0.019**	Not excluded
dNLR	125	1.254 (0.975-1.612) 0.077	114	1.223 (0.957-1.563) 0.107	1)
LMR	125	**0.689 (0.564-0.841) <0.001**	114	**0.608 (0.422-0.877) 0.008**	Not excluded
PLR	125	**1.005 (1.001-1.008) 0.008**	114	**1.282 (1.001-1.643) 0.049**	4)**
Platelet count	138	1.001 (0.998-1.003) 0.634	126	1.069 (0.860-1.327) 0.548	1)
Absolute leucocyte count	138	1.004 (0.923-1.092) 0.933	126	1.093 (0.865-1.380) 0.434	1)
Absolute lymphocyte count	125	**0.659 (0.458-0.948) 0.025**	114	0.089 (0.606-1.080) 0.150	1) and 4)**
Relative lymphocyte count	125	**0.959 (0.928-0.990) 0.011**	114	**0.758 (0.583-0.985) 0.038**	3) and 4)
Absolute neutrophile count	125	1.032 (0.943-1.130) 0.492	114	1.141 (0.912-1.426) 0.248	1)
Relative neutrophile count	124	1.023 (0.993-1.054) 0.137	113	1.175 (0.904-1.527) 0.229	1)
Absolute eosinophile count	124	0.602 (0.067-5.394) 0.650	113	0.959 (0.713.1.288) 0.779	1)
Relative eosinophile count	124	0.929 (0.761-1.133) 0.467	113	0.875 (0.650-1.177) 0.376	1)
Absolute monocyte count	125	2.637 (0.911-7.632) 0.074	114	1.200 (0.939-1.534) 0.145	1)
Relative monocyte count	125	**1.178 (1.069-1.299) <0.001**	114	**1.332 (1.049-1.692) 0.019**	3) and 4)***
LDH	53	**1.008 (1.002-1.013) 0.010**	50	1.277 (0.759-2.148) 0.358	1) and 2)
CRP	138	**1.065 (1.026-1.105) <0.001**	126	**1.457 (1.214-1.747) <0.001**	Not excluded

Exclusion criteria:

1) Not significant in univariate or multivariate Cox regression analysis.
2) Patient number n<100.
3) Relative blood values, if there are absolute values provided.

4) High pairwise Pearson correlation (r ≥0.7) between blood values. In this case, the weaker parameter regarding exclusion criteria 1-3 was dropped.<br>* Note: Single blood value in multivariate Cox regression analysis with following co-variates: Age, AJCC-stage, capsule invasion, adjuvant interferon-α, size of biggest SLN metastasis.<br><br>** High correlation with NLR.<br>*** High correlation with LMR.<br>Note: Significant results are **bold.**

Table S2b: Overview of results of all blood variables in univariate and multivariate Cox regression analysis for overall survival (OS) with applied exclusion criteria.

**Blood values** (continuous parameter)	**Univariate Cox regression analysis** n	HR (95% CI) p-value	**Multivariate Cox regression analysis*** n	HR (95% CI) p-value	**Exclusion criteria**
NLR	124	1.174 (0967-1.426) 0.105	114	**1.396 (1.004-1.941) 0.047**	4)**
dNLR	124	1.287 (0.945-1.753) 0.109	114	**1.410 (1.024-1.942) 0.035**	Not excluded
LMR	124	0.818 (0.642-1.042) 0.103	114	0.769 (0.504-1.256) 0.326	1)
PLR	125	1.003 (0.998-1.007) 0.306	114	1.148 (0.804-1.639) 0.446	1)
Platelet count	136	1.000 (0.997-1.003) 0.977	126	1.103 (0.818-1.488) 0.518	1)
Absolute leucocyte count	136	1.044(0.943-1.155) 0.407	126	**1.334 (1.022-1.742) 0.034**	Not excluded
Absolute lymphocyte count	124	0.777 (0.482-1.251) 0.299	114	0.935 (0.639-1.370) 0.732	1)
Relative lymphocyte count	124	0.966 (0.926-1.008) 0.109	114	0.710 (0.492-1.023) 0.066	1)
Absolute neutrophile count	124	1.081 (0.975-1.200) 0.140	114	**1.404 (1.086-1.815) 0.010**	Not excluded
Relative neutrophile count	123	1.027 (0.987-1.068) 0.187	113	1.399 (0.951-2.057) 0.088	1)
Absolute eosinophile count	123	0.060 (0.002-1.757) 0.103	113	9.864 (0.564-1.323) 0.500	1)
Relative eosinophile count	123	0.745 (0.548-1.012) 0.059	113	0.764 (0.501-1.165) 0.211	1)
Absolute monocyte count	124	1.538 (0.358-6.620) 0.563	114	1.125 (0.808-1.569) 0.485	1)

Relative monocyte count	124	1.050 (0.915-1.206) 0.486	114	0.939 (0.679-1.299) 0.704	1)
LDH	51	**1.027 (1.014-1.041) <0.001**	48	**4.877 (1.550-15.342) 0.007**	2)
CRP	136	1.019 (0.958-1.084 0.548	126	1.216 (0.928-1.594) 0.157	1)

Exclusion criteria:

5) Not significant in univariate or multivariate Cox regression analysis.
6) Patient number n<100.
7) Relative blood values, if there are absolute values provided.
8) High pairwise Pearson correlation (r ≥0.7) between blood values. In this case, the weaker parameter regarding exclusion criteria 1-3 was dropped.

* Note: Single blood value in multivariate Cox regression analysis with following co-variates: Age, AJCC-stage, capsule invasion, adjuvant interferon-α, size of biggest SLN metastasis.
** High correlation with dNLR.
Note: Significant results are **bold.**

Table S3. Univariate Cox regression analysis with covariates for recurrence-free survival and overall survival.

	**Univariate Cox analysis**	
Variable<br><br>(Reference bold)	**Recurrence-free survival**<br><br>HR (95% CI) p-value	**Overall survival**<br><br>HR (95% CI) p-value
Age<br>(n=55; n=83)<br>>65 years vs **≤65 years**	**1.985 (1.261-3.125) 0.003**	**3.060 (1.657-5.650) <0.001**
AJCC-stage<br>(n=36, n=32, n=70, n=0)<br>IIIB vs **IIIA**<br>IIIC vs **IIIA**<br>IIID vs **IIIA**	2.247 (0.992-5.087) 0.052<br>**4.303 (2.114-8.759) <0.001**<br>/	1.656 (0.589-4.658) 0.339<br>**2.685 (1.111-6.490) 0.028**<br>/
Capsule invasion<br>(n=15; n=122)<br>yes vs **no**	1.677 (0.858-3.277) 0.130	**4.394 (2.120-9.109) <0.001**
Adjuvant interferon-α		

(n=45; n=92)<br>yes vs **no**	0.692 (0.422-1.133)<br>0.143	**0.329 (0.156-0.692)**<br>**0.003**
Size of biggest SLN metastasis<br>(n=66; n=62)<br>> 1mm vs ≤ **1mm**	**1.849 (1.139-3.004)**<br>**0.013**	**3.444 (1.674-7.086)**<br>**<0.001**
Gender<br>(n=67; n=71)<br>female vs **male**	0.721 (0.458-1.136)<br>0.158	0.672 (0.364-1.239)<br>0.203
Ulceration<br>(n=64, n=74)<br>yes vs **no**	**2.390 (1.505-3.794) <0.001**	**2.044 (1.108-3.770)**<br>**0.022**
Mitotic rate<br>(n=95; n=5)<br>>1/mm² vs **≤ 1/mm²**	0.260 (0.036-1.881) 0.182	0,045 (0.000-64.245) 0.403
Number of affected SLN<br>(n=108; n=22; n=8)<br>2 vs **1**<br>3 vs **1**	0.784 (0.401-1.534)<br>0.477<br>1.921 (0.873-4.227)<br>0.104	0.493 (0.175-1.385)<br>0.179<br>0.621 (0.149-2.579)<br>0.512
Satellite metastases<br>(n=8; n=130)<br>yes vs **no**	0.683 (0.249-1.873)<br>0.459	0.968 (0.298-3.138)<br>0.956
CLND<br>(n=72; n=66)<br>yes vs **no**	**1.710 (1.072-2.729)**<br>**0.024**	1.356 (0.728-2.525)<br>0.337
Note: Significant results are **bold.**		

Table S4. Univariate and multivariate Cox regression analysis with multiple blood value cut-offs of NLR, LMR and CRP for recurrence-free survival.

**Blood value combinations**			
**Univariate Cox analysis**		**Multivariate Cox analysis ***	
Event number	HR (95% CI) p-value	Event number	HR (95% CI) p-value
NLR (≥3.5) + CRP (>3.0)		NLR (≥3.5) + CRP (>3.0)	
1 (n=45) vs **0** (n=71) events	**2.673 (1.616-4.421) <0.001**	1 (n=39) vs **0** (n=67) events	**2.370 (1.352-4.156) 0.003**
2 (n=9) vs **0** (n=71) events	**4.131 (1.872-9.117) <0.001**	2 (n=8) vs **0** (n=67) events	**4.838 (2.009-11.652) <0.001**
1 (n=45) vs **2** (n=9) events	0.647 (0.298-1.402) 0.270	1 (n=39) vs **2** (n=8) events	0.490 (0.200-1.203) 0.119
LMR (<3.5) + CRP (>3.0)		LMR (<3.5) + CRP (>3.0)	
1 (n=57) vs **0** (n=51) events	**3.365 (1.870-6.054) <0.001**	1 (n=53) vs **0** (n=48) events	**3.239 (1.710-6.135) <0.001**
2 (n=17) vs **0** (n=51) events	**6.386 (3.119-13.076) <0.001**	2 (n=13) vs **0** (n=48) events	**7.700 (3.436-17.255) <0.001**
1 (n=57) vs **2** (n=17) events	**0.527 (0.289-9.60) 0.036**	1 (n=53) vs **2** (n=13) events	**0.421 (0.212-0.835) 0.013**
NLR (≥3.5) + LMR (<3.5)		NLR (≥3.5) + LMR (<3.5)	
1 (n=36) vs **0** (n=63) events	**2.133 (1.217-3.738) 0.008**	1 (n=32) vs **0** (n=59) events	**2.216 (1.211-4.055) 0.010**
2 (n=26) vs **0** (n=63) events	**2.614 (1.463-4.671) 0.001**	2 (n=23) vs **0** (n=59) events	**2.160 (1.113-4.189) 0.023**
1 (n=36) vs **2** (n=26) events	0.816 (0.448-1.488) 0.507	1 (n=32) vs **2** (n=23) events	1.026 (0.521-2.020) 0.941
NLR (≥3.5) + LMR (<3.5) + CRP (>3.0)		NLR (≥3.5) + LMR (<3.5) + CRP (>3.0)	
1 (n=41) vs **0** (n=48) events	**3.054 (1.612-5.788) <0.001**	1 (n=39) vs **0** (n=45) events	**3.398 (1.709-6.755) <0.001**
2 (n=28) vs **0** (n=48) events	**4.167 (2.148-8.083) <0.001**	2 (n=23) vs **0** (n=45) events	**3.515 (1.647-7.500) 0.001**
3 (n=8) vs **0** (n=48) events	**6.059 (2.456-14.945) <0.001**	3 (n=7) vs **0** (n=45) events	**7.690 (2.789-21.202) <0.001**
1 (n=41) vs **3** (n=8) events	0.504 (0.218-1.165) 0.109	1 (n=39) vs **3** (n=7) events	0.442 (0.173-1.128) 0.087
2 (n=28) vs **3** (n=8) events	0.688 (0.293-1.617) 0.391	2 (n=23) vs **3** (n=7) events	0.457 (0.167-1.251) 0.128

*Single blood value in multivariate Cox regression analysis with following covariates: Age, AJCC-stage, capsule invasion, adjuvant Interferon-α, size of biggest SLN metastasis.

Note: Significant results are **bold.**

Supplementary figure

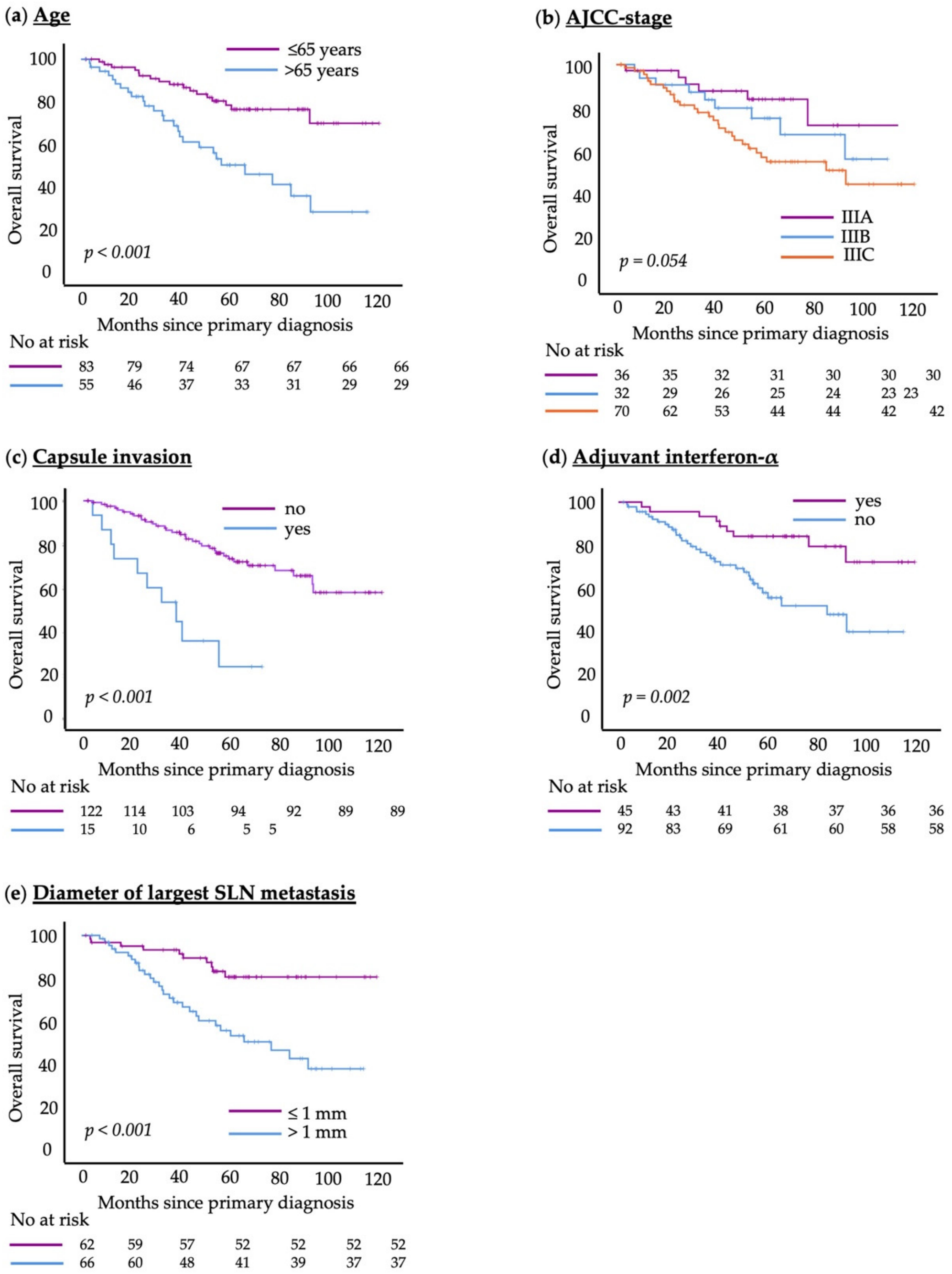

Figure S1. Kaplan-Meier survival curves showing overall survival for covariates of the multivariate Cox regression model: (**a**) patient age; (**b**) AJCC-stage; (**c**) capsule invasion of sentinel lymph node (SLN) metastasis; (**d**) adjuvant interferon-α therapy; (**e**) diameter of largest SLN metastasis. The log-rank test was used to compare between groups; $p < 0.05$ was considered significant.
